# Engineering Metal–Organic Framework-Biopolymer-Based Hydrogels for Therapeutic Delivery

**DOI:** 10.1021/acsmaterialsau.6c00029

**Published:** 2026-05-14

**Authors:** Talia A. Shmool, Néis Lartigue, Xu Liu, Jinjie Zhu, Maungo R. Poomore, Robert D. Hunter, Paul F. McKay, Jesús Barrio, Theoni K. Georgiou, Robin J. Shattock

**Affiliations:** † Department of Chemical Engineering, 4615 Imperial College London, South Kensington Campus, London SW7 2AZ, U.K.; ‡ Department of Materials, Imperial College London, South Kensington Campus, London SW7 2AZ, U.K.; § Department of Infectious Disease, Imperial College London, South Kensington Campus, London SW7 2AZ, U.K.; ∥ Institute of Veterinary Pharmacology and Toxicology, University of Zurich, Winterthurerstrasse 260, CH-8057 Zurich, Switzerland

**Keywords:** hydrogels, drug delivery, biopolymers, metal−organic frameworks, advanced characterization

## Abstract

Biopolymer-based hydrogels are attractive therapeutic carriers, offering tunable physicochemical properties and therapeutic release kinetics. Major limitations include low rheological strength, poor physical and thermal stability, limited swelling, and achieving controlled therapeutic delivery. To address these challenges, a library of innovative metal–organic framework (MOF)-biopolymer-based hydrogels was developed. The MOFs, zeolitic imidazole framework-8 (ZIF-8), and zinc adeninate framework (ZAF) were integrated into chitosan/alginate (C/A) and chitosan/gelatin (C/G) hydrogels, at increasing chitosan content. The MOF-hydrogels presented distinct immunoglobulin G (IgG) release rates and greater rheological strengths, swelling capabilities, and thermostabilities compared to the MOF lacking hydrogels. The MOF-C/A-hydrogels showed higher rheological strengths compared to the MOF-C/G-hydrogels. The ZIF-8-hydrogels presented greater rheological strengths, yet lower thermostabilities, and higher IgG release rates compared to the ZAF-hydrogels. This is attributed to the greater flexibility of ZAF, containing bulky adenine groups, which could lead to steric hindrance and limited zinc ion–dipole interactions. Holistically, exploiting ion–dipole, electrostatic, and hydrogen bonding interactions between the MOFs and biopolymers enabled therapeutic release rate control and balanced the typical trade-off between hydrogel swelling and rheological strength. The MOF-hydrogels offer adaptable platforms, advancing the design of next-generation MOF-biopolymer-based carriers for target applications.

## Graphical abstract

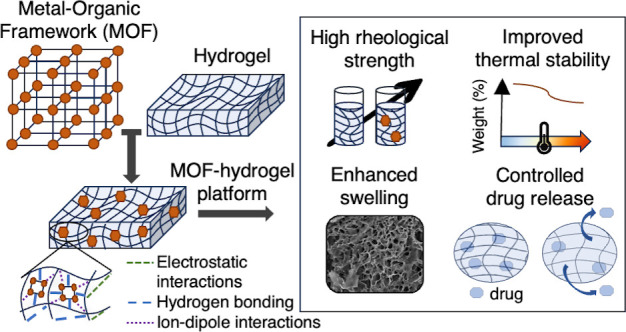

## Introduction

Therapeutic carriers for controlled delivery applications can be rationally engineered to provide predictable therapeutic release kinetics over a specified period, offering extended therapeutic half-life, improved bioavailability, and suppressed aggregation and degradation.^1^ In particular, hydrogels, water-swollen three-dimensional structures, are attractive delivery platforms, as the physical and chemical properties of hydrogels can be tuned to precisely control the release of therapeutics over space and time and allow for long-term release.^2,3^ The biopolymers chitosan, alginate, and gelatin have been extensively employed in the development of controlled hydrogel delivery platforms, including wound dressings.^2,4–6^ Chitosan-based hydrogels have been shown to provide increased mechanical strength,^7,8^ biodegradability,^8–10^ and allow for controlled and sustained drug release; however, typically present low solubility in aqueous solutions, limiting pharmaceutical applications.^11^ Alginate comprised hydrogels offer highly tunable surface modification for localized drug delivery;^12,13^ yet, present limited mechanical and thermal stability,^14–16^ critical for achieving sustained and prolonged drug release. Gelatin inclusive hydrogels have demonstrated versatile physicochemical properties^17,18^ that can be precisely adjusted to increase drug loading capacity; however, these exhibit poor physical stability, low mechanical strength, and rapid degradation.^19,20^ Notably, chitosan/alginate and chitosan/gelatin hydrogels have been shown as versatile delivery platforms of enhanced structural and thermal stability^21,22^ that allow for modulation of hydrogel mechanical strength and swelling to achieve desirable controlled release profiles.^23–25^ Nonetheless, limitations remain in terms of achieving spatiotemporal control over therapeutic release and balancing the trade-off between increased swelling capability and rheological strength of hydrogels.^6,26,27^

More recently, metal–organic frameworks (MOFs), constructed via metal nodes and organic linkers, have demonstrated opportunities in hydrogel delivery system design.^28–30^ MOFs offer high surface areas, tunable pore sizes, and stimuli-responsive properties, enabling greater drug loading and fine-control over drug release rates.^29,31–33^ Notably, few studies have engineered zeolitic imidazole framework-8 (ZIF-8) modified hydrogels for drug delivery applications,^34–36^ and have demonstrated that MOF integration can yield systems of greater mechanical strengths, structural stabilities, and tailored drug release profiles.^31,37,38^ Additionally, ZIFs, a subclass of MOFs, allow for the construction of multivariate structures with targeted functionalities.^39,40^ For example, the ZIF-8 framework has been modified with adenine, yielding a biocompatible framework built via the self-assembly of zinc adeninate macrocycles.^41^

Herein, we were inspired to develop a design approach for engineering MOF-biopolymer-based hydrogels comprised of chitosan/alginate (C/A) and chitosan/gelatin (C/G), overcoming the limitations of existing hydrogel platforms. We aim to create MOF-hydrogels offering enhanced thermal stabilities and rheological strengths, allow for modulation of swelling, and controlled therapeutic release. We embed ZIF-8 and zinc adeninate framework (ZAF) in C/A-hydrogels and C/G-hydrogels of increasing chitosan content, 0–100%m/m, referring to the dry mass of alginate, chitosan, and gelatin. Notably, ZAF offers greater loading capacity and structural flexibility compared to ZIF-8,^41^ attractive for fine-tuning the degree of swelling of the hydrogel. ZIF-8 is highly microporous, and possesses a greater surface area compared to ZAF, which could allow for higher structural stability and rheological strength. Additionally, ZIF-8 and ZAF have been shown to offer high water stability,^32,41,42^ particularly attractive for integration in biopolymer-based hydrogels for enhanced structural and thermal stability. The selected components have each been previously reported to exhibit favorable biocompatibility in biomedical contexts.^15,41,43–45^ We investigate the influence of each MOF and chitosan content on the physicochemical, rheological, and morphological properties, and the release behavior of the MOF-hydrogel formulations. We select a fixed MOF loading of 5%m/m, referring to the dry mass, as a representative and employed composition in MOF-hydrogels, enabling direct comparison across formulations.^38,46^ It has been shown that higher MOF content could enable greater mechanical strength, yet reduced swelling; while lower MOF content could yield MOF-hydrogels offering higher therapeutic release rates.^47–49^ Notably, ZIF-8 and ZAF embedded in C/A-hydrogels and C/G-hydrogels have yet to be systematically investigated, and we aim to gain insight into the fundamental ion-dipole, hydrogen bonding and electrostatic interactions in the MOF-hydrogels influencing system attributes. We perform visual tests to systematically determine the effect of each MOF and biopolymer on the gelation processes of the developed formulation. We examine the rheological strengths, morphologies, swelling behaviors, and thermal stabilities of the MOF-hydrogels, utilizing rheology experiments, scanning electron microscopy (SEM), swelling tests, and thermogravimetric analysis (TGA), respectively. Finally, we evaluate the release profiles of the model therapeutic immunoglobulin G (IgG) from the MOF-hydrogels utilizing an enzyme-linked immunosorbent assay (ELISA).

## Results

### Visual Assessment of MOF-Hydrogels

Overall, chitosan content above 30%m/m in MOF-C/A formulations and above 50%m/m in MOF-C/G formulations was required for MOF-hydrogel formation. Specifically, ZIF-8 and ZAF in 30C/70A, 40C/60A, 50C/50A, 60C/40A, 70C/30A, 80C/20A, 90C/10A, 50C/50G, 60C/40G, 70C/30G, 80C/20G, 90C/10G, and 100C, and ZAF-20C/80A, demonstrated MOF-hydrogel formation at 25 °C (Figure 1, Figure S1). Notably, ZIF-8 in 20C/80A, and ZIF-8 and ZAF in 100A, 10C/90A, 100G, 10C/90G, 20C/80G, 30C/70G, and 40C/60G failed to yield MOF-hydrogel formation, nor upon heating to 40 °C (Figure 1, Figure S1).

**Figure 1:**
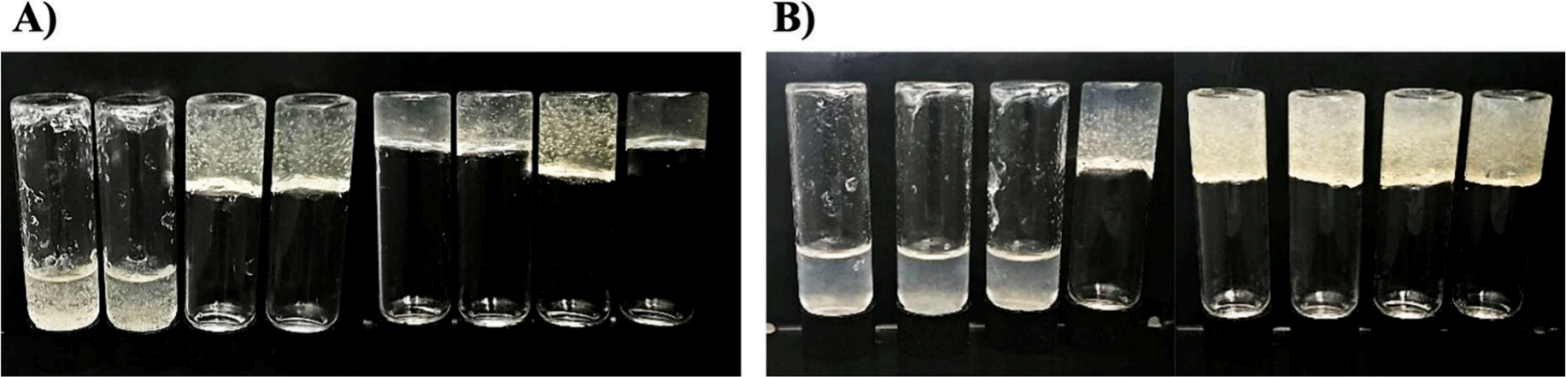
Photographs of MOF-hydrogels, from left to right. **A)** ZIF-8-40C/60G, ZAF-40C/60G, ZIF-8-50C/50G, ZAF-50C/50G, ZIF-8-60C/40G, ZAF-60C/40G, ZIF-8-70C/30G and ZAF-70C/30G. **B)** ZIF-8–100C/90A, ZAF-100C/90A, ZIF-8-20C/80A, ZAF-20C/80A, ZIF-8-70C/30A, ZAF-70C/30A, ZIF-8-80C/20A and ZAF-80C/20A. For additional images see Figure S1.

### Rheological Properties of MOF-Hydrogel Formulations

We evaluated the rheological properties of the formulations which visually demonstrated MOF-hydrogel formation and found that for each system G′ was higher than G″ (Figure 2, Figure S2), indicating MOF-hydrogel formation. ZIF-8-100C and ZAF-100C exhibited higher rheological strengths compared to 100C lacking MOFs (G′ = 113, 403, and 89 Pa, respectively), demonstrating that MOF incorporation serves to increase hydrogel rheological strength (Table S1). Additionally, increasing chitosan content was linked to greater MOF-hydrogel rheological strength, at a threshold chitosan content of 80%m/m for both ZIF-8 and ZAF in C/A-hydrogels and C/G-hydrogels. Specifically, ZIF-8-70C/30A, ZAF-80C/20A, ZIF-8-80C/20G, and ZIF-8-70C/30G displayed the highest rheological strengths (G′ = 2835 and 2619 Pa, 585 and 416 Pa, respectively); and ZAF-20C/80A, ZIF-8-40C/60A, ZAF-50C/50G and ZAF-60C/40G showed the lowest rheological strengths (G′ = 150 Pa, 218 Pa, 41 and 60 Pa, respectively). Overall, we found that ZIF-8 and ZAF in C/A-hydrogels showed significantly greater rheological strengths compared to C/G-hydrogels, and ZIF-8-hydrogels exhibited higher rheological strengths compared to ZAF-hydrogels, for both C/A and C/G formulations above 50%m/m chitosan.

**Figure 2:**
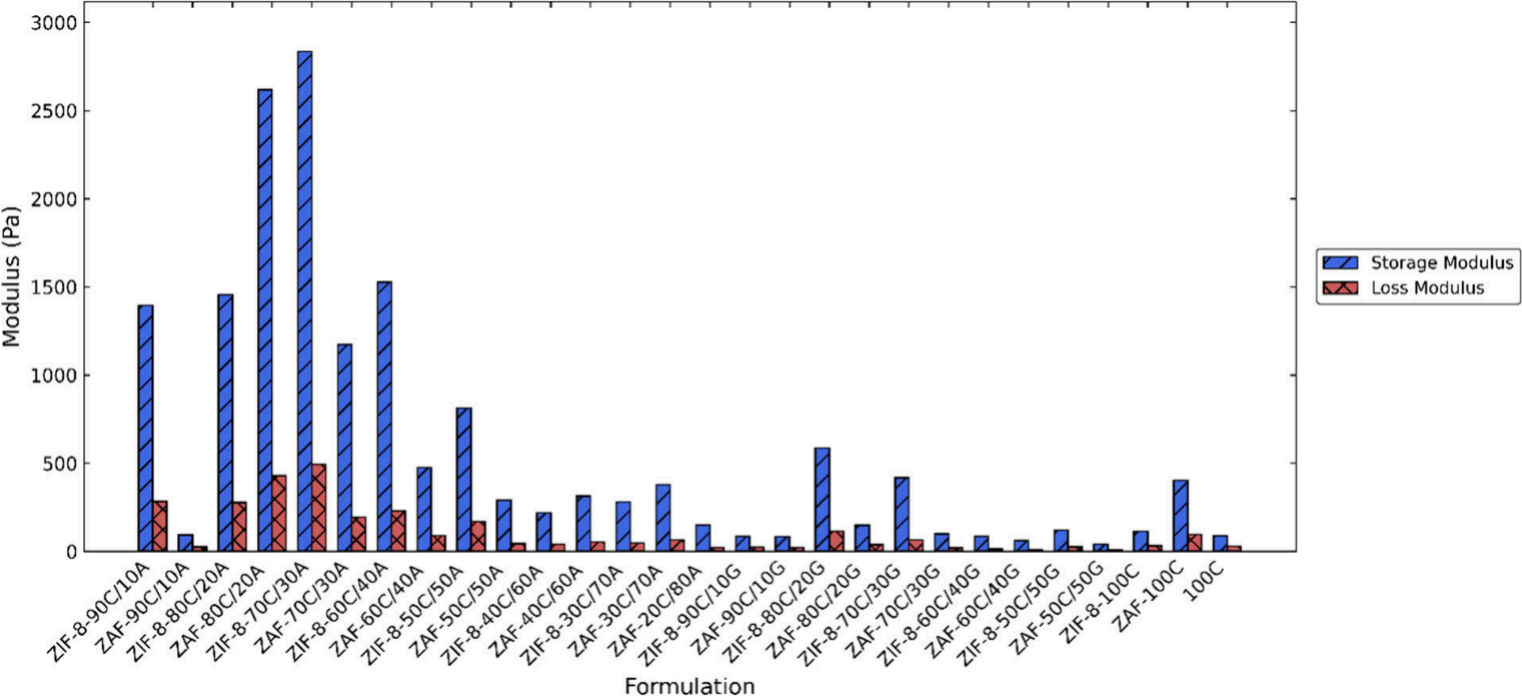
G′ and G″ at 37 °C, 0.5% strain, and 1 rad·s^–1^ angular frequency for 100C, and ZIF-8 and ZAF in C/A and C/G hydrogels.

### Swelling Tests of MOF-Hydrogel Formulations

We evaluated the mass swelling factor (*SF*_*m*_) of ZIF-8 and ZAF in C/G and C/A formulations at 0.17, 0.33, 0.5, 1, 2, and 20 h (Figure 3, Table S2, Table S3), and found that *SF*_*m*_ increased significantly directly following immersion in PBS, exceeding 6 mg·mg^–1^ at 0.17 h for each formulation. The ZAF-C/A-hydrogels displayed the highest swelling, lower for ZIF-8-C/G-hydrogels and further reduced for ZAF-C/G and ZIF-8-C/A formulations. The highest *SF*_*m*_ was found for ZAF-70C/30A and ZAF-80C/20A at 20 h, and the lowest for ZAF-90C/10A at 0.17 h. Notably, MOF-hydrogels of reduced chitosan content, including ZAF-20C/80A, ZIF-8-40C/60A, and ZIF-8 and ZAF in 30C/70A, exhibited lower rheological strengths and *SF*_*m*_ over time. Additionally, increasing chitosan content from 50 to 80%m/m served to raise both the *SF*_*m*_ values and rheological strengths of the MOF-hydrogels. In the absence of MOF, 100C showed a *SF*_*m*_ of 32.1 at 0.17 h, and a substantial decrease over time, exhibiting the lowest *SF*_*m*_ at 20 h. The sharp decrease in the *SF*_*m*_ of 100C, as opposed to reaching a plateau, along with the visual disintegration of 100C observed over time may be linked to rapid hydrogel degradation.^50^

**Figure 3:**
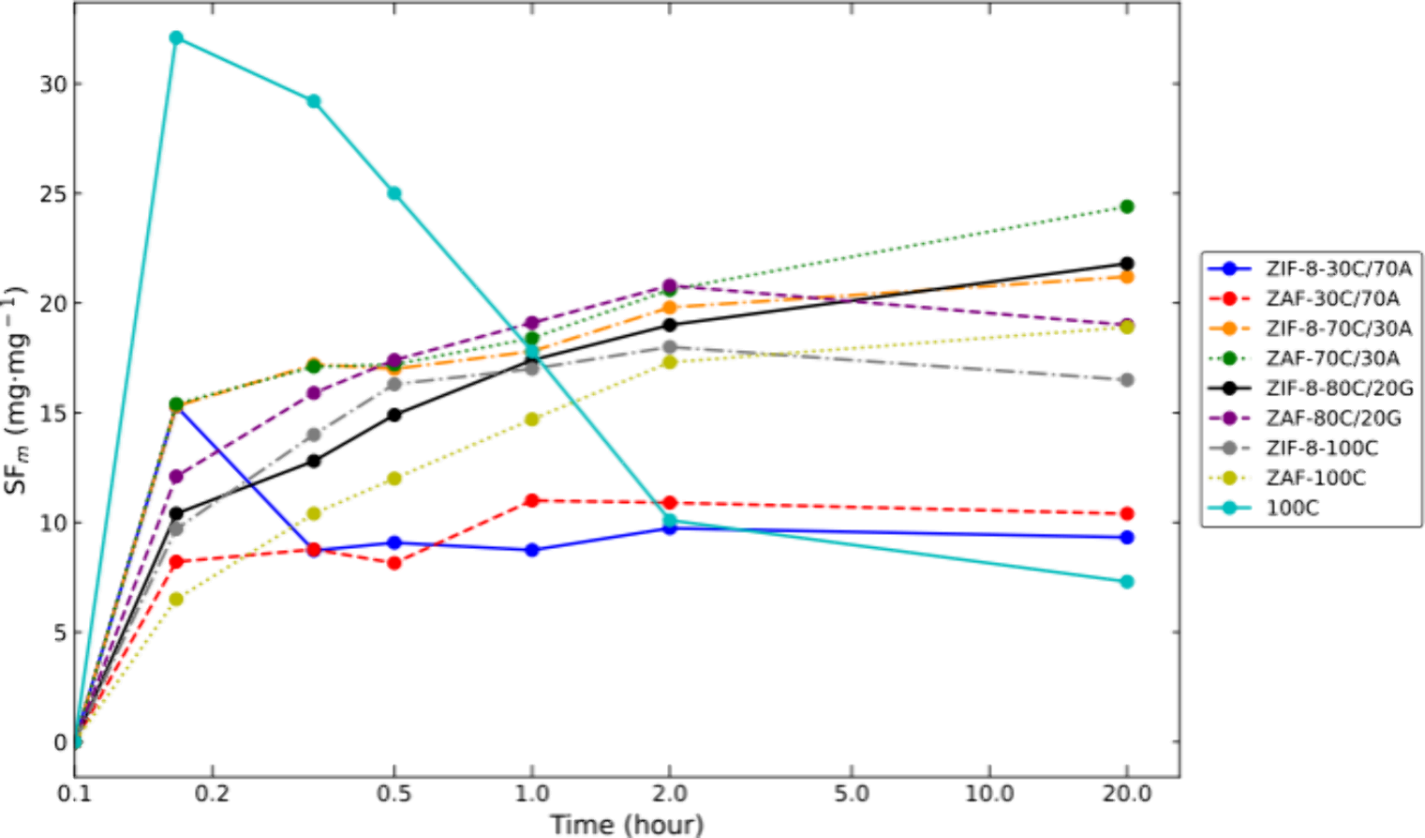
Mass swelling factors (*SF*_*m*_) as a function of time. The time axis is logarithmic. For *SF*_*m*_ values see Tables S2 and S3.

### Morphologies of MOFs and MOF-Hydrogels

SEM was performed to examine the morphologies of ZIF-8 and ZAF and of the MOF-hydrogels of greatest rheological strengths, including ZIF-8 and ZAF in 80C/20A and 70C/30A, and ZIF-8-60C/40A. For contrast, we also examined ZIF-8 and ZAF in 80C/20G and 100C, ZAF-60C/40A, and 100C lacking MOFs, which displayed relatively lower G′ values.

SEM images revealed that ZIF-8 exhibited uniform particle sizes of 100 nm in agreement with previous works;^51,52^ and ZAF displayed greater plate-like particles (Figure S3). Overall, we observed smooth and porous networks for the MOF-hydrogels (Figure 4, Figure S4). Specifically, ZIF-8 and ZAF in 100C and C/G formulations exhibited relatively more porous networks compared to ZIF-8 and ZAF in C/A formulations. For ZIF-8 and ZAF in C/A and C/G formulations above 80%m/m chitosan, more uniform porous networks were observed. Notably, the 100C formulations lacking MOFs exhibited the least dense networks and overall indicated that MOF integration reduces pore size.

**Figure 4:**
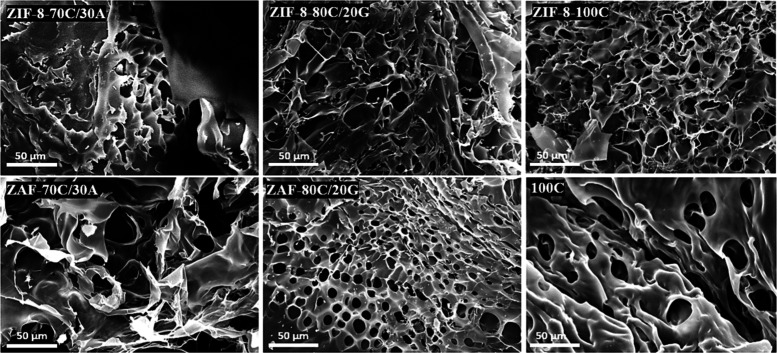
SEM images showing the cross-section of select MOF-hydrogels. For additional images see Figures S3 and S4.

### Thermal Stabilities of MOF-Hydrogels

TGA measurements were performed to evaluate the thermal stabilities of ZIF-8-60C/40A, ZIF-8-70C/30A, ZIF-8 and ZAF in 80C/20A, ZIF-8-70C/30G, ZIF-8 and ZAF in 80C/20G (Figure 5). These formulations displayed the greatest rheological strengths and *SF*_*m*_, and thus selected for further evaluation. We also examined 100C lacking MOFs, and ZIF-8-100C and ZAF-100C to investigate the effect of each MOF on formulation thermal stability.

**Figure 5:**
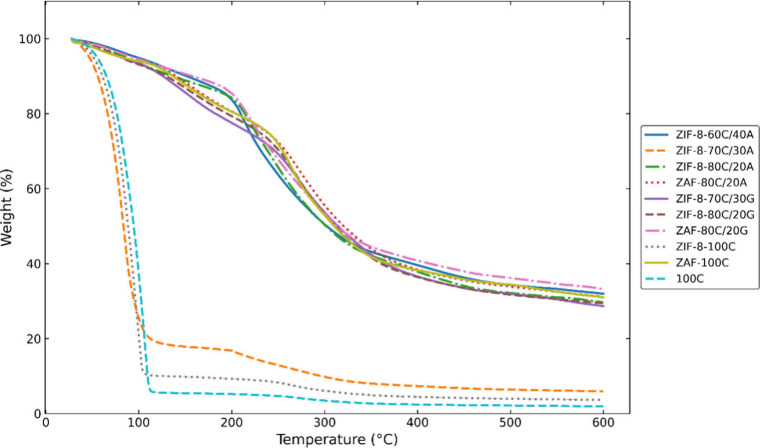
TGA curves for the formulations, measured between 30 and 600 °C at 5 °C per minute.

100C lacking MOFs displayed the highest and most rapid weight loss, with 94% and 98% weight loss at 115 and 600 °C, respectively. ZAF-hydrogels displayed lower weight loss compared to the ZIF-8-hydrogels for both the C/A and C/G homologue formulations. Additionally, ZIF-8-100C and ZAF-100C displayed 90% and 7% weight loss at 115 °C, highlighting the role of ZAF in modulating and raising formulation thermal stability synergistically upon C/A and C/G integration. Moreover, ZIF-8 and ZAF in C/G formulations exhibited relatively high thermal stabilities, and ZAF-80C/20G showed 6.5% weight loss at 115 °C and the lowest weight loss, 67%, at 600 °C. Notably, of the systems examined, ZIF-8-70C/30A, of greatest rheological strength also displayed the highest weight loss of 80% at 115 °C.

### Therapeutic Release Behavior

ZIF-8 and ZAF in 80C/20A and 70C/30A, and ZIF-8-60C/40A, displayed the greatest rheological strengths and swelling, and thus selected for examination of IgG release rate. Typically, IgG is used as a model protein for therapeutic release studies, as IgG can be rapidly and accurately quantified in the release media employing standardized anti-immunoglobulin assays. We also investigated 100C lacking MOFs, ZIF-8-100C, and ZAF-100C to evaluate the influence of the MOFs on therapeutic release kinetics.

The 100C-hydrogels lacking MOFs showed the highest release rate, 11577 ng·mL^–1^ IgG at 24 h (Figure 6A, cumulative% release displayed based on the ng·mL^–1^ measurement data). ZIF-8-100C, ZIF-8-60C/40A, and ZAF-100C released 12478, 12134, and 10859 ng·mL^–1^ IgG, respectively, at 68 h, also displaying linear release rates. Area under the curve (AUC) analysis showed that 100C presented the highest overall release during the 68 h period (Figure 6B). ZIF-8 and ZAF in 70C/30A and 80C/20A exhibited lower release rates, with ZAF-80C/20A releasing 284.90 ng·mL^–1^ and 1708.2 ng·mL^–1^ IgG at 24 and 68 h, respectively. The lowest AUC values were observed for ZAF-80C/20A and ZAF-70C/30A, consistent with more sustained and lower release rates. Overall, ZAF-hydrogel formulations presented relatively lower release rates compared to ZIF-8-hydrogel formulations. Notably, C/A-hydrogels above 70%m/m chitosan, displayed greater rheological strengths and the lowest IgG release rates, which could suggest reduced MOF-hydrogel degradation. One-way ANOVA followed by Tukey’s post hoc test indicated statistically significant differences primarily observed between the 100C formulations and several MOF-hydrogel formulations. Specifically, 100C exhibited significantly higher cumulative IgG release compared to ZIF-8-70C/30A (*p*-value = 0.034), ZAF-70C/30A (*p*-value = 0.031), ZIF-8-80C/20A (*p*-value = 0.036), and ZAF-80C/20A (*p*-value = 0.027). Holistically, MOF integration served to yield systems of distinct release profiles and the MOF-hydrogels show potential for controlled and sustained therapeutic release applications.

**Figure 6:**
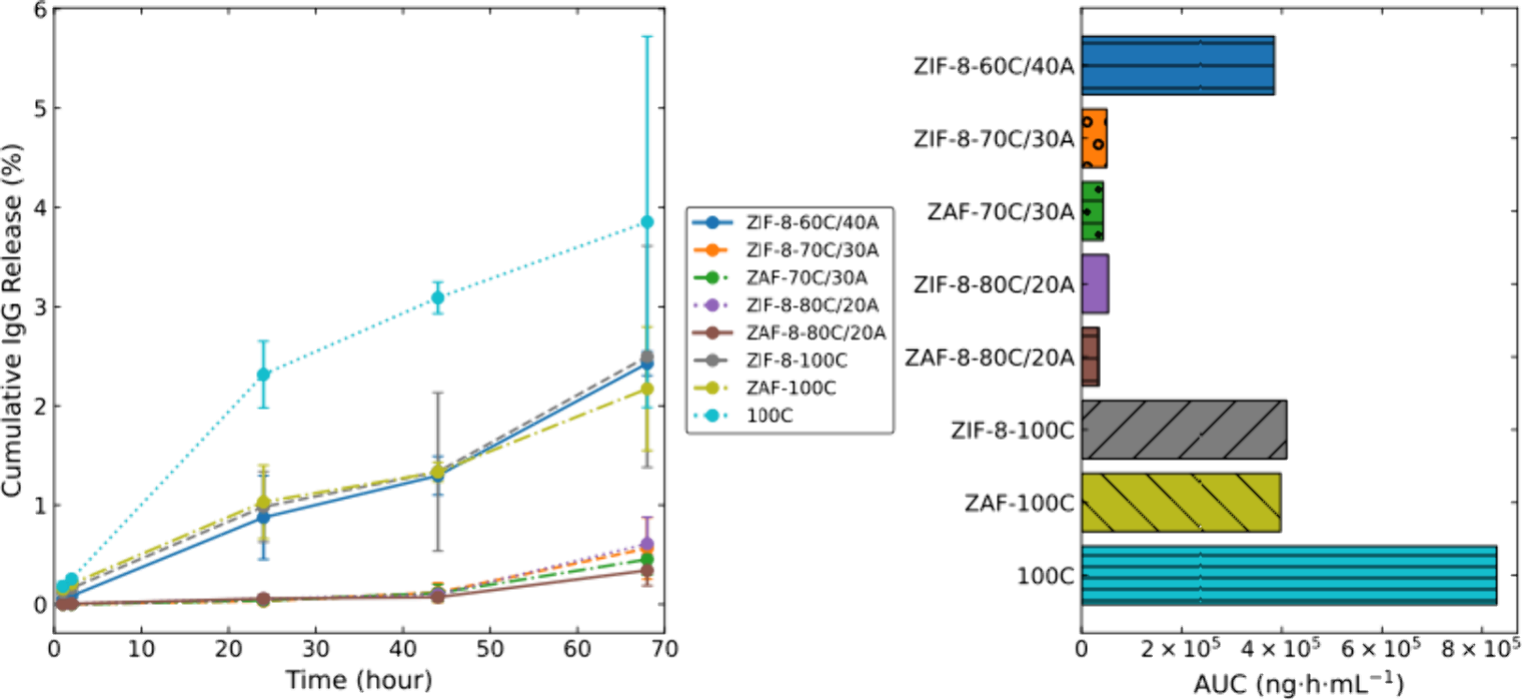
**A)** Cumulative release of IgG (%) from MOF-hydrogel formulations over 68 h. **B)** Cumulative release of IgG (AUC). Error bars represent the standard deviation of mean, *n* = 3 samples. For complete ELISA data see Table S4.

## Discussion

To optimally engineer MOF-biopolymer-based hydrogels, the influence of ZIF-8 and ZAF on the physicochemical, rheological and morphological properties of the C/A and C/G hydrogels were systematically examined. We suggest that the zinc ions and imidazole rings of ZIF-8 and ZAF may serve to facilitate ion-dipole, electrostatic and hydrogen bonding interactions with the acetamide groups of chitosan,^53^ and the carboxylate groups of alginate^54^ and gelatin, yielding MOF-hydrogels formation. We propose that the stronger intermolecular and ion-dipole interactions serve to raise the rheological strengths, thermal stabilities, swelling capabilities, and provide enhanced control over IgG release rates for ZIF-8 and ZAF in 100C, C/G and C/A-hydrogels, compared to each formulation lacking MOFs. Distinctly, the adenine groups of ZAF could contribute additional hydrogen bonding interactions with the hydroxy amino groups of chitosan,^55^ increasing the thermal stabilities of the ZAF-hydrogels compared to ZIF-8-C/A and ZIF-8-C/G formulations.^56^ ZIF-8 possesses a structure based on coordination bonds and is relatively more rigid compared to the hydrogen-bonded ZAF framework.^41^ The greater flexibility of ZAF and bulkier adenine groups, compared to imidazole rings, could result in steric hindrance^57^ limiting strong zinc ion-dipole bonding interactions.^58^ This would explain the relatively lower IgG release rates of the ZAF-C/A-hydrogels, and reduced rheological strengths of ZAF-C/G and ZAF-C/A formulations, below 50%m/m chitosan. Notably, structural characterization and spectroscopic analysis, including solid-state NMR and Fourier Transform Infrared Spectroscopy could provide further insight into the proposed molecular interactions and network architecture of the MOF-hydrogels.^46,59^ We suggest that the cationic amine groups of chitosan could facilitate strong electrostatic interactions with the anionic carboxylate groups of alginate,^60^ and hydrogen bonding interactions in the hydroxyl groups of chitosan and alginate may serve to increase the rheological strength and thermal stability and reduce the degree of porosity of the MOF-C/A-hydrogels, as we observed. We consider that 100C lacking MOFs and C/A formulations of reduced chitosan content, likely exhibit limited electrostatic, ion-dipole and hydrogen bonding interactions, resulting in relatively lower rheological strengths for the 30C/70A and 40C/60A formulations. This also explains the lack of MOF-hydrogel formation for ZIF-8 and ZAF in 100A, 10C/90A, and ZIF-8-20C/80A. Additionally, the amine and hydroxyl groups of chitosan could facilitate electrostatic and hydrogen bonding interactions with the hydroxyl and carboxylate groups of gelatin.^61,62^ In contrast to alginate, the weaker intermolecular interactions promoted via gelatin and limited confinement effects would explain the reduced rheological strengths of the C/G-hydrogels compared to the C/A formulations, and lack of MOF-hydrogel formation for ZIF-8 and ZAF formulations including 60–100%m/m gelatin.

Typically, stronger biopolymer-based hydrogels exhibit limited swelling capabilities;^63,64^ yet herein, increasing chitosan content, between 60 and 80%m/m, in the MOF-C/A and MOF-C/G formulations resulted in MOF-hydrogels of greater rheological strengths and swelling capabilities. This is attributed to a balance between steric hindrance, electrostatic, ion-dipole and hydrogen bonding interactions in the MOFs and C/A and C/G networks serving to increase rheological strength via confinement effects,^65,66^ and altering the porosity and hydrophilic nature of the MOF surface to enable greater swelling. This is consistent with the postulation that increasing chitosan content results in stronger intermolecular interactions between the IgG and the MOF-C/A-hydrogels.^67^ Moreover, the relatively more uniform porous structures of ZIF-8 and ZAF in 100C, and 100C lacking MOF could be linked to the higher IgG release rate of these formulations^68^ compared to ZIF-8 and ZAF in 70C/30A and 80C/20A, also presenting dense networks. We propose that the IgG release rate is influenced by diffusion and MOF-polymer interactions, as per preliminary release kinetic analysis using the Korsmeyer-Peppas model; and dense MOF-hydrogel networks could exhibit reduced release rates, consistent with diffusion behavior.^69,70^ Further modeling and experimental validation are required to confirm the dominant release mechanisms.

It is worth noting that increasing the concentration of ZIF-8 has been shown to reduce the swelling capabilities of hydrogel systems.^38,49^ The hydrophobic nature of ZIF-8 and the imidazole-based framework of ZAF likely reduces water absorption and the swelling capabilities of the MOF-hydrogels. As such, increasing the concentration of ZIF-8 and ZAF could serve to restrict hydrogel swelling and simultaneously reduce therapeutic release rates. Thus, systematically exploring MOF content optimization would be beneficial in future work. Finally, the structural stability of ZIF-8-hydrogels and ZAF-hydrogels in aqueous media should be considered, as hydrolysis and degradation could influence the rheology behavior and therapeutic release rate over time.^71^ Further studies could systematically assess MOF-hydrogel degradation under physiological and pathological conditions to support mechanistic interpretations and long-term therapeutic performance.

## Conclusions

This study demonstrates the rational design of MOF-biopolymer-based hydrogels, offering innovative platforms for controlled therapeutic delivery applications. We developed a library of ZIF-8 and ZAF in C/A-hydrogels and C/G-hydrogels providing enhanced rheological strengths, swelling capabilities, and thermal stabilities compared to hydrogels lacking MOFs. ZIF-8 and ZAF in C/A and C/G formulations, below 30 and 50%m/m chitosan, respectively, failed to yield MOF-hydrogels. The MOF-C/A-hydrogels showed significantly higher rheological strengths compared to the MOF-C/G-hydrogels, attributed to greater electrostatic and hydrogen bonding interactions promoted via alginate. Notably, MOFs in C/A-hydrogels and C/G-hydrogels mitigated the typical trade-off between rheological strength and swelling capability of biopolymer-based hydrogels. ZIF-8-hydrogels, including above 50%m/m chitosan, exhibited the greatest rheological strengths and ZAF-hydrogels displayed the highest thermal stabilities and lower IgG release rates. Furthermore, ZIF-8 and ZAF in C/A formulations containing 70 and 80%m/m chitosan demonstrated the lowest IgG release rates compared to the 100C-hydrogel lacking MOFs and ZIF-8 and ZAF in 100C-hydrogels, which also displayed more uniform porous structures and higher IgG release rates. The engineered MOF-hydrogel formulations offer tunable features for a broad range of therapeutic delivery applications. We believe that with greater exploration of MOF-hydrogel formulations, our fundamental understanding of MOF-biopolymer interactions and the opportunities of MOF-based technologies for pharmaceutical applications will be realized.

## Experimental Section

### Materials

Chitosan (medium molecular weight, 190–310 kDa, degree of deacetylation of ≥ 75%), sodium alginate (M/G ratio of 0.43), gelatin from bovine skin, propionic acid, phosphate buffered saline (PBS), Tween-20 and purified IgG from human serum were purchased from Sigma-Aldrich, Dorset, UK. Goat antihuman kappa, Goat antihuman IgG-HRP (1:4000 dilution in assay buffer) and lambda light chain specific antibodies were purchased from Southern Biotech, Birmingham, USA. KPL SureBlue TMB substrate and H_2_SO_4_ Stop Solution were purchased from Insight Biotechnology, Wembley, UK. All materials were used without further purification.

### Synthesis of MOFs

ZIF-8 and ZAF were synthesized as previously described.^41,72^ Briefly, ZAF was synthesized by heating an equimolar ratio of adenine, 2-methyleimidazole, and zinc acetate dihydrate in isopropanol (200 mL) at 70 °C for 24 h. It was then centrifuged at 10 000 rpm for 10 min, the supernatant was discarded, and the solid powder was washed with hot isopropanol at 50 °C, centrifuged, and dried at 80 °C overnight. ZIF-8 was synthesized by dissolving zinc nitrate hexahydrate (0.3 g) in methanol (11.3 g), followed by the addition of 2-methylimidazole (0.66 g) in methanol (11.3 g) at 25 °C. The mixture was vigorously stirred, then centrifuged, and washed with methanol twice. The resulting ZIF-8 powder was dried overnight at 80 °C. Powder X-ray diffraction confirmed the successful synthesis of ZIF-8 and ZAF (Figure S5).

### Preparation of MOF-Hydrogel Formulations

For the MOF-hydrogels, a series of chitosan (C) and alginate (A), and chitosan (C) and gelatin (G) formulations were prepared. The biopolymers were mixed at the specified amounts (Table 1) and dissolved in a 2.5% v/v propionic acid in deionized water solution (3 mL), found as the optimal solvent for this study, yielding the most homogeneous MOF-hydrogels (Figure S6). 5%m/m ZIF-8 and ZAF were integrated in each formulation, as previously described.^38^ Additionally, 100%m/m chitosan (100C), alginate (100A) and gelatin (100G) excluding MOFs were prepared. Each formulation was mixed via vortex (Cole-Parmer, Illinois, USA) for 5 min at 25 °C.

**Table 1:** Formulations of MOF-Hydrogels Comprising Chitosan (C) and Alginate (A); and C and Gelatin (G), Expressed as %m/m^a^

Formulation	Chitosan (%m/m)	Alginate (%m/m)	Gelatin (%m/m)
**0C/100A**	0	100	0
**10C/90A**	10	90	0
**20C/80A**	20	80	0
**30C/70A**	30	70	0
**40C/60A**	40	60	0
**50C/50A**	50	50	0
**60C/40A**	60	40	0
**70C/30A**	70	30	0
**80C/20A**	80	20	0
**90C/10A**	90	10	0
**100C**	100	0	0
**0C/100G**	0	0	100
**10C/90G**	10	0	90
**20C/80G**	20	0	80
**30C/70G**	30	0	70
**40C/60G**	40	0	60
**50C/50G**	50	0	50
**60C/40G**	60	0	40
**70C/30G**	70	0	30
**80C/20G**	80	0	20
**90C/10G**	90	0	10

a Each formulation includes 5%m/m ZIF-8 and ZAF, and %m/m refers to dry mass of the MOFs, alginate, chitosan, and gelatin as specified.

### Powder X-ray Diffraction (XRD)

To confirm successful MOF synthesis, powder XRD patterns (0.016° scan step size) were obtained using a PANalytical X’PERT PRO powder X-ray diffractometer with a Cu Kα source operating at 45 kV and 30 mA.

### Lyophilization

Formulations were stored under −20 °C for 24 h, then lyophilized using a Virtis Benchtop Pro Freeze-Dryer (SP Scientific, New York, USA) for 48 h to obtain the lyophilized MOF-hydrogel formulations. These were examined via SEM and swelling tests.

### Rheology Experiments

Rheology measurements were performed using a HR-10 Discovery Hybrid rheometer (Waters TA Instruments, New Castle, USA) to determine the rheological strengths of the MOF-hydrogels. The experiments were conducted at 37 °C, to reflect the rheological behavior of the MOF-hydrogels under physiologically relevant conditions. The storage modulus (G′) and loss modulus (G″) were determined at a constant strain of 0.5% and at varying frequencies of 0.1 to 100 rad·s^–1^.

### Scanning Electron Microscopy (SEM)

SEM was performed using the JSM-6010LA InTouchScope Multiple touch panel scanning electron microscope (JEOL Ltd., Tokyo, Japan) to examine the morphology of the MOF-hydrogels. The MOF-hydrogels were fixed to an aluminum stub using adhesive carbon tape and sputter coated with gold under vacuum. Images were recorded using secondary electron imaging at an accelerating voltage of 20 kV.

### Swelling Tests

Swelling tests of the lyophilized MOF-hydrogels were conducted in PBS as previously described.^21,73^ Notably, the tests were conducted at 25 °C over 20 h, to assess the swelling capabilities of the MOF-hydrogels over an extended time period, while limiting MOF-hydrogel degradation. The mass swelling factor (*SF*_*m*_)^74,75^ of each MOF-hydrogel was evaluated using eq 1:

1 $$SF_{m}=\frac{m_{s}-m_{d}}{m_{d}}$$

where *m*_*d*_ is the mass of the dry MOF-hydrogel prior to dissolution in PBS and *m*_*s*_ is the mass of the swollen MOF-hydrogel. The MOF-hydrogels were withdrawn from the aqueous solution at 0.17, 0.33, 0.5, 1, 2, and 20 h. Excess PBS on the surface of each MOF-hydrogel was removed using Whatman filter paper and the swollen state mass of each was measured using a microbalance (Ohaus, New Jersey, US) at 25 °C.

### Thermogravimetric Analysis (TGA)

The thermostabilities of the MOF-hydrogels were evaluated using a TGA8000 instrument (PerkinElmer, Shelton, Connecticut, USA). Experiments were performed under a nitrogen environment using a purge flow of 40 mL·min^–1^, between 30 and 600 °C, with a heating rate of 5 °C·minute^–1^_._

### Human IgG ELISA Method

To assess IgG release rates, a defined amount of human IgG was directly mixed with the MOF-hydrogels at 25 °C. The formulations were submerged into physiological media and then samples were removed periodically for measurement, using a standardized human IgG ELISA, of the amount of human IgG released from the MOF-hydrogel. Briefly, 96-well high binding MaxiSorp plates (Nunc) were coated with 100 μL·well^–1^ Goat antihuman kappa and lambda light chain specific antibodies, at a 1:3 000 dilution in PBS. Following overnight incubation at 4 °C, the plates were washed using PBS-T (1x PBS + 0.05% Tween-20), blocked for 1 h at 37 °C employing assay buffer (PBS supplemented with 1% BSA and 0.05% Tween-20), washed, and the diluted samples and standard were added in assay buffer. Plates were then incubated for 1 h at 37 °C, washed and the secondary detection antibody was added (Goat antihuman IgG-HRP, 1:4000 dilution in assay buffer). Following a final 1 h incubation at 37 °C, plates were washed with PBS-T and developed with 50 μL·well^–1^ of KPL SureBlue TMB substrate. The reaction was halted following 5 min by adding 50 μL·well^–1^ of 1 M H_2_SO_4_ and the absorbance was read at 450 nm on a FLUOstar Omega spectrophotometer (BMG Labtech, Ortenberg, Germany). Cumulative% release was calculated by converting ELISA-derived IgG concentrations to released mass at each time point, accounting for sampling volume and dilution, followed by cumulative summation. Values were normalized to the total theoretical IgG loading, determined from the initial IgG concentration and MOF-hydrogel formulation volume. Statistical analyses were performed using GraphPad Prism (version 10.04.1627). Release data were obtained from independent experimental replicates, n = 3 formulations per time point, and corrected for dilution factors. Statistical comparisons between formulations were conducted using one-way ANOVA followed by Tukey’s multiple comparisons test to evaluate differences in cumulative IgG release profiles. Statistical significance was defined as *p*-value < 0.05.

## Supplementary Material



## Data Availability

The authors declare that the data supporting the findings of this study are available in this paper and its Supporting Information. Should any raw data be needed in another format, these will be available from the corresponding author upon reasonable request.

## Notes

Photographs of the formulations in propionic acid, ZIF-8-20C/80G, ZAF-20C/80G, ZIF-8-30/70G, ZAF-30C/70G, ZIF-8-80C/20G, ZAF-80C/20G, ZIF-8-90C/10G, ZAF-90C/10G, ZIF-8-50C/50A, ZAF-50C/50A, ZIF-8-60C/40A, ZAF-60C/40A, 100C lacking MOFs, ZIF-8-100C and ZAF-100C; evolution of storage modulus (G′) and loss modulus (G″) as a function of the applied frequency for the MOF-hydrogel formulations and the G′ and G″ at 37 °C, 0.5% strain, and 1 rad·s^–1^ angular frequency for ZIF-8 and ZAF in C/A-hydrogels, C/G-hydrogels, and 100C-hydrogels; swelling mass factor (*SF*_*m*_) measured at 10, 20, 30, 60, 120, and 1200 min for MOF-C/A-hydrogels and MOF-C/G-hydrogels; crystal SEM images of 100C, ZIF-8, ZAF, and ZIF-8 and ZAF-80C/20A, 60C/40A; data for the ELISA quantification of IgG release (ng·mL^–1^) from ZIF-8 and ZAF-hydrogel formulations over 68 h; powder X-ray diffraction and simulated pattern from the Crystallographic Information File (CIF) of ZIF-8 and ZAF, confirming the successful synthesis of ZIF-8 and ZAF; photographs of 60C/40A-hydrogels and 50C/50G-hydrogels in propionic, decanoic, oxalic, and malonic acids, l-arginine, and ammonium nitrate, demonstrating differences in formulation homogeneity (PDF)
