# Supplementary material for: Engineering Metal–Organic Framework-Biopolymer-Based Hydrogels for Therapeutic Delivery
Source: ACS Mater Au. 2026 May 14;6(4):831–40. doi: 10.1021/acsmaterialsau.6c00029 (PMC13352276; doi:10.1021/acsmaterialsau.6c00029)
Supplement: Supplementary file 1 [file mg6c00029_si_001.pdf]

Supporting Information

Engineering Metal-Organic Framework-Biopolymer-Based Hydrogels for Therapeutic Delivery

Talia A. Shmool,[a‡]* Néis Lartigue,[a,d‡] Xu Liu,[b] Jinjie Zhu,[a] Maungo R. Poomore,[a] Robert D. Hunter,[a] Paul F. McKay,[c] Jesús Barrio,[a] Theoni K. Georgiou,[b] Robin J. Shattock[c]

[a] Department of Chemical Engineering, Imperial College London, South Kensington Campus, London SW7 2AZ, UK

[b] Department of Materials, Imperial College London, South Kensington Campus, London SW7 2AZ, UK

[c] Department of Infectious Disease, Imperial College London, South Kensington Campus, London SW7 2AZ, UK

[d] Institute of Veterinary Pharmacology and Toxicology, University of Zurich, Winterthurerstrasse 260, CH-8057 Zurich, Switzerland

* E-mail: t.shmool20@imperial.ac.uk; Telephone: +44 (0)20 7589 5111

‡ Authors contributed equally

Visual tests

A) **B)**

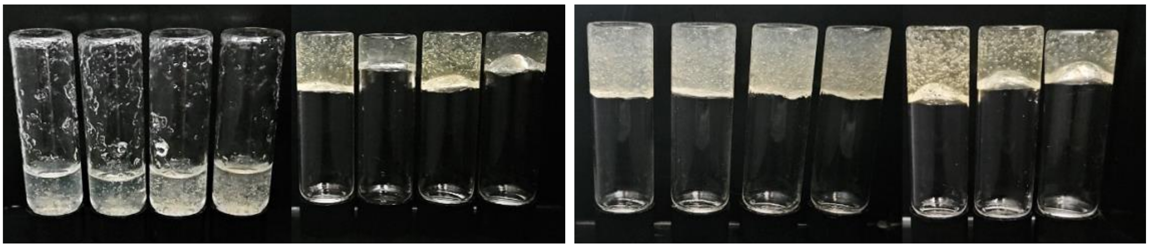

Figure S1. Photographs of the formulations in propionic acid, from left to right, **A)** ZIF-8-20C/80G, ZAF-20C/80G, ZIF-8-30C/70G, ZAF-30C/70G, ZIF-8-80C/20G, ZAF-80C/20G, ZIF-8-90C/10G and ZAF-90C/10G; **B)** ZIF-8-50C/50A, ZAF-50C/50A, ZIF-8-60C/40A, ZAF-60C/40A, 100C lacking MOFs, ZIF-8-100C and ZAF-100C.

Rheology experiments

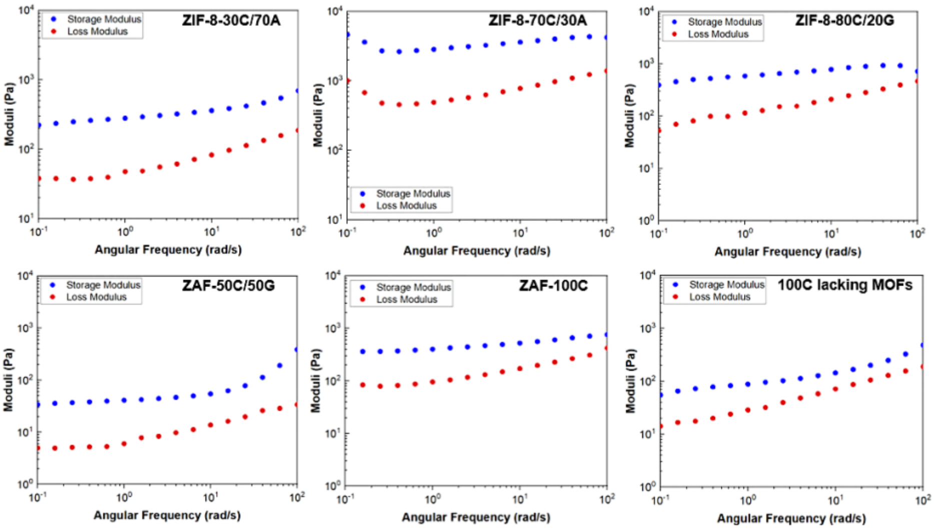

Figure S2. Evolution of storage modulus (G′) and loss modulus (G″) as a function of the applied frequency for the MOF-hydrogel formulations.

Table S1. G′ and G″ at 37 °C, 0.5% strain and 1 rad·s^{-1} angular frequency for ZIF-8 and ZAF in C/A-hydrogels, C/G-hydrogels and 100C-hydrogels.

MOF-hydrogel formulation	G′ (Pa)	G″ (Pa)
**ZIF-8-90C/10A**	1395	283.2
**ZAF-90C/10A**	91.78	25.78
**ZIF-8-80C/20A**	1455	277.0
**ZAF-80C/20A**	2619	428.4
**ZIF-8-70C/30A**	2835	492.8
**ZAF-70C/30A**	1173	193.5
**ZIF-8-60C/40A**	1526	229.2
**ZAF-60C/40A**	475.8	89.60
**ZIF-8-50C/50A**	812.1	169.4
**ZAF-50C/50A**	291.1	43.46
**ZIF-8-40C/60A**	218.4	40.02
**ZAF-40C/60A**	313.1	53.94
**ZIF-8-30C/70A**	279.9	47.66
**ZAF-30C/70A**	379.0	65.36
**ZAF-20C/80A**	149.6	21.38
**ZIF-8-90C/10G**	84.77	22.90
**ZAF-90C/10G**	83.70	21.39
**ZIF-8-80C/20G**	585.4	114.3
**ZAF-80C/20G**	148.6	37.92
**ZIF-8-70C/30G**	416.3	64.89
**ZAF-70C/30G**	98.22	18.94
**ZIF-8-60C/40G**	85.65	14.61
**ZAF-60C/40G**	60.34	10.31
**ZIF-8-50C/50G**	120.1	25.31
**ZAF-50C/50G**	41.19	6.079

**ZIF-8-100C**	113.4	29.87
**ZAF-100C**	403.3	96.15
**100C**	88.68	28.69

Swelling tests

Table S2. Swelling mass factor (SF_m) measured at 10, 20, 30, 60, 120, and 1200 minutes for MOF-C/A-hydrogels. SF_m for each MOF-hydrogel formulation at 0 minutes was 0 mg·mg^{-1}.

Formulation	Time (minute)	SF_m (mg·mg^{-1})
**ZAF-20C/80A**	10	9.64
	20	7.15
	30	7.01
	60	6.70
	120	7.41
	1200	7.57
**ZAF-30C/70A**	10	8.21
	20	8.77
	30	8.15
	60	11.0
	120	10.9
	1200	10.4
**ZAF-40C/60A**	10	12.6
	20	10.3
	30	10.5
	60	8.39
	120	8.01
	1200	6.79
**ZAF-50C/50A**	10	11.7
	20	13.6
	30	15.7
	60	19.0
	120	20.4
	1200	22.0
**ZAF-60C/40A**	10	20.1
	20	19.3
	30	19.6
	60	20.1
	120	19.5
	1200	18.8
**ZAF-70C/30A**	10	15.4
	20	17.1
	30	17.2
	60	18.4

	120	20.6
	1200	24.4
**ZAF-80C/20A**	10	13.1
	20	15.1
	30	16.7
	60	19.4
	120	21.4
	1200	24.4
**ZAF-90C/10A**	10	6.41
	20	10.5
	30	13.5
	60	16.2
	120	17.2
	1200	17.6
**ZAF-100C**	10	6.50
	20	10.4
	30	12.0
	60	14.7
	120	17.3
	1200	18.9
**ZIF-8-30C/70A**	10	15.3
	20	8.72
	30	9.08
	60	8.74
	120	9.74
	1200	9.32
**ZIF-8-40C/60A**	10	15.4
	20	11.7
	30	12.1
	60	12.9
	120	12.9
	1200	13.8
**ZIF-8-50C/50A**	10	13.8
	20	16.2
	30	18.6
	60	20.3
	120	21.3
	1200	22.9
**ZIF-8-60C/40A**	10	18.6
	20	17.1
	30	17.0
	60	18.8
	120	18.7

	1200	21.0
**ZIF-8-70C/30A**	10	15.3
	20	17.2
	30	17.0
	60	17.8
	120	19.8
	1200	21.2
**ZIF-8-80C/20A**	10	16.4
	20	17.8
	30	17.4
	60	16.1
	120	17.3
	1200	16.5
**ZIF-8-90C/10A**	10	10.9
	20	14.3
	30	15.1
	60	15.9
	120	15.8
	1200	16.1
**ZIF-8-100C**	10	9.70
	20	14.0
	30	16.3
	60	17.0
	120	18.0
	1200	16.5

Table S3. Swelling mass factor (SF_m) measured at 10, 20, 30, 60, 120, and 1200 minutes for MOF-C/G-hydrogels and 100C lacking MOFs. SF_m for each MOF-hydrogel formulation at 0 minutes was 0 mg·mg^{-1}.

**Formulation**	**Time (minute)**	SF_m **(mg·mg^{-1})**
**ZAF-50C/50G**	10	6.79
	20	7.99
	30	8.39
	60	9.87
	120	11.7
	1200	14.3
**ZAF-60C/40G**	10	6.28
	20	8.09
	30	9.03
	60	10.8
	120	12.6
	1200	16.7

**ZAF-70C/30G**	10	8.78
	20	11.0
	30	12.8
	60	15.1
	120	18.4
	1200	22.4
**ZAF-80C/20G**	10	12.1
	20	15.9
	30	17.4
	60	19.1
	120	20.8
	1200	19.0
**ZAF-90C/10G**	10	8.33
	20	11.1
	30	12.6
	60	16.4
	120	18.8
	1200	21.4
**ZIF-8-50C/50G**	10	18.1
	20	20.1
	30	19.9
	60	20.4
	120	21.4
	1200	20.4
**ZIF-8-60C/40G**	10	9.89
	20	11.0
	30	12.1
	60	13.4
	120	15.7
	1200	18.4
**ZIF-8-70C/30G**	10	15.7
	20	17.4
	30	19.3
	60	21.1
	120	20.9
	1200	22.4
**ZIF-8-80C/20G**	10	10.4
	20	12.8
	30	14.9
	60	17.4
	120	19.0
	1200	21.8
**ZIF-8-90C/10G**	10	14.6

	20	17.8
	30	19.7
	60	20.1
	120	19.6
	1200	17.6
**100C lacking MOF**	10	32.1
	20	29.2
	30	25.0
	60	17.8
	120	10.1
	1200	7.3

SEM experiments

The SEM images were collected with Zeiss LEO Gemini 1525 SEM. ZIF-8 exhibited a uniform particle size of ~100 nm, and the typical rhombic dodecahedral morphology. ZAF displayed plate-like particles with an average size of approximately 4 μm.

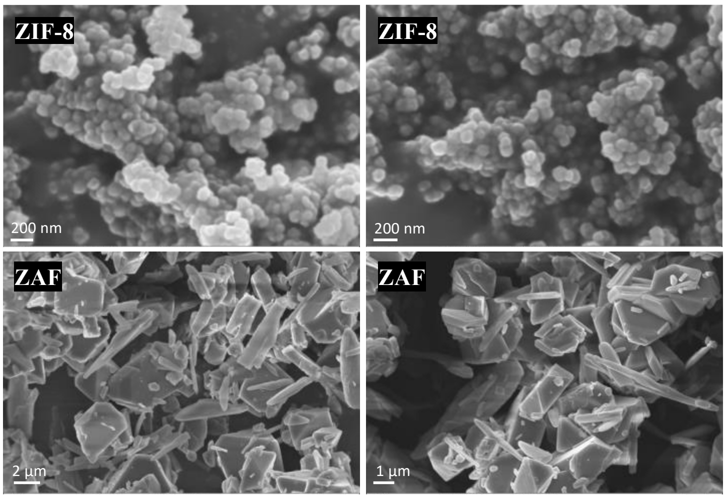

Figure S3. SEM images of ZIF-8 and ZAF.

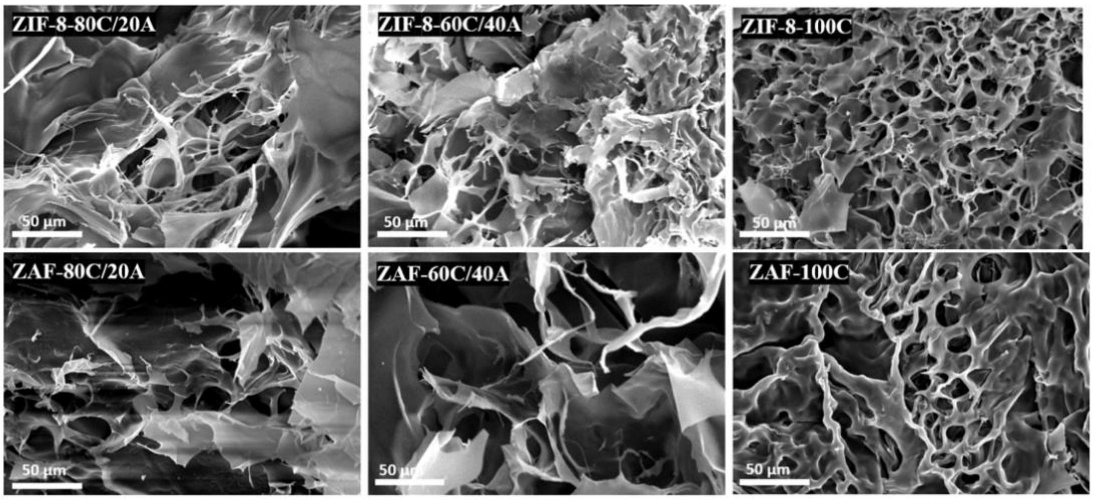

Figure S4. SEM images showing the cross-section of the MOF-hydrogels.

IgG release rate evaluation

Table S4. ELISA quantification of IgG release ($ng \cdot mL^{-1}$) from the formulations over 68 hours. Data represents the mean standard deviation (SD) for three independent measurements, with dilution accounted for (×300). Time points include 1, 2, 24, 44 and 68 hours, highlighting the variations in release rates between the formulations.

Formulation	Time (hour)	Mean release ($ng \cdot mL^{-1}$)	SD
**ZIF-8-60C/40A**	1	252.300	101.400
	2	420.75	58.344
	24	4372.1	2109.6
	44	6479.9	973.13
	68	12134	613.79
**ZIF-8-70C/30A**	1	0	0
	2	14.800	25.634
	24	159.40	163.86
	44	592.10	488.98
	68	2816.2	1554.3
**ZAF-70C/30A**	1	0.0000	0.0000
	2	0.0000	0.0000
	24	187.40	72.874
	44	562.20	432.89
	68	2260.3	786.28
**ZIF-8-80C/20A**	1	14.400	3.1749
	2	21.700	7.1077
	24	265.50	114.15
	44	494.30	258.31
	68	3047.8	1347.6
**ZAF-80C/20A**	1	13.900	12.370
	2	28.600	34.698
	24	284.90	224.46
	44	356.40	177.25
	68	1708.2	773.80
**ZIF-8-100C**	1	602.40	266.44
	2	811.80	112.85
	24	4908.8	1778.3
	44	6675.0	3991.9
	68	12478	5580.1
**ZAF-100C**	1	759.90	149.71
	2	1029.3	92.408
	24	5154.4	1847.2
	44	6661.3	490.16
	68	10859	3118.3

**100C**	1	900.60	232.06
	2	1274.4	124.34
	24	11577	1681.7
	44	15443	796.98
	68	19268	9354.8

Synthesis of MOFs

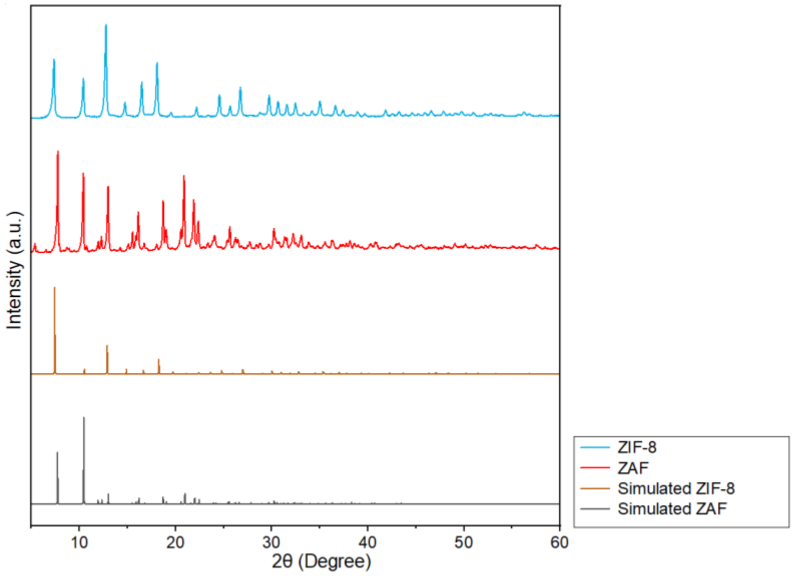

Figure S5. Powder X-ray diffraction and simulated pattern from Crystallographic Information File (CIF) of ZIF-8 and ZAF, confirming the successful synthesis of ZIF-8 and ZAF.

Visual tests

Initially, for MOF-hydrogel preparation, chitosan (C)/alginate (A) and chitosan (C)/gelatin (G) of increasing chitosan content 0-100 %m/m, were dissolved in 2 %v/v acetic acid in deionised water. Visual tests demonstrated that the formulations lacked homogeneity, and thus C/A and C/G compositions were dissolved in formic, propionic, butanoic, valeric, decanoic, oxalic, malonic, adipic acids, L-arginine, and ammonium nitrate (Figure S6) at 0.33 mmol·mL^{-1}. The solvent selected for MOF-hydrogel preparation was propionic acid, providing the most visually homogeneous MOF-hydrogels.

A) **B)**

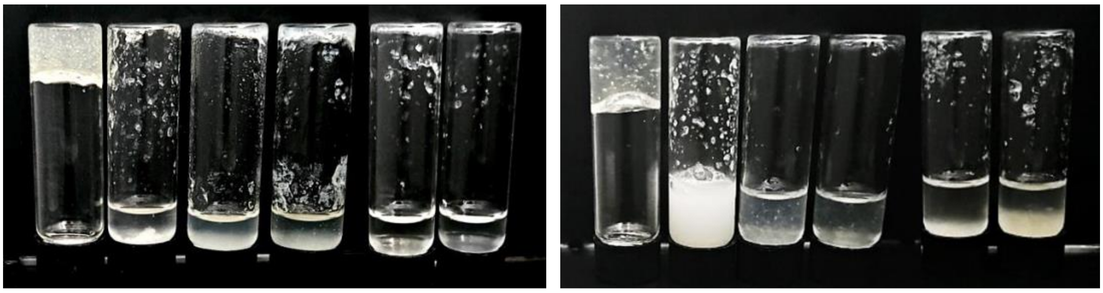

Figure S6. Photographs of **A)** 60C/40A hydrogels in propionic, decanoic, oxalic, malonic acids, L-arginine, and ammonium nitrate (left to right); and **B)** 50C/50G hydrogels in propionic, decanoic, oxalic, malonic acids, L-arginine, and ammonium nitrate (left to right).
